# Thermodynamics of Gas–Liquid Colloidal Equilibrium States: Hetero-Phase Fluctuations

**DOI:** 10.3390/e21121189

**Published:** 2019-12-03

**Authors:** Leslie V. Woodcock

**Affiliations:** Department of Physics, University of Algarve, 8005-139 Faro, Portugal; lvwoodcock@ualg.pt

**Keywords:** hetero-phase fluctuation, percolation transition, supercritical mesophase, liquid state, argon

## Abstract

Following on from two previous JETC (Joint European Thermodynamics Conference) presentations, we present a preliminary report of further advances towards the thermodynamic description of critical behavior and a supercritical gas-liquid coexistence with a supercritical fluid mesophase defined by percolation loci. The experimental data along supercritical constant temperature isotherms (T ≥ T_c_) are consistent with the existence of a two-state mesophase, with constant change in pressure with density, rigidity, (dp/dρ) _T_, and linear thermodynamic state-functions of density. The supercritical mesophase is bounded by 3rd-order phase transitions at percolation thresholds. Here we present the evidence that these percolation transitions of both gaseous and liquid states along any isotherm are preceded by pre-percolation hetero-phase fluctuations that can explain the thermodynamic properties in the mesophase and its vicinity. Hetero-phase fluctuations give rise to one-component colloidal-dispersion states; a single Gibbs phase retaining 2 degrees of freedom in which both gas and liquid states with different densities percolate the phase volume. In order to describe the thermodynamic properties of two-state critical and supercritical coexistence, we introduce the concept of a hypothetical homo-phase of both gas and liquid, defined as extrapolated equilibrium states in the pre-percolation vicinity, with the hetero-phase fractions subtracted. We observe that there can be no difference in chemical potential between homo-phase liquid and gaseous states along the critical isotherm in mid-critical isochoric experiments when the meniscus disappears at T = T_c_. For T > T_c_, thermodynamic states comprise equal mole fractions of the homo-phase gas and liquid, both percolating the total phase volume, at the same temperature, pressure, and with a uniform chemical potential, stabilised by a positive finite interfacial surface tension.

## 1. Introduction

We have known for several years, from computer experiments on model square-well molecular fluids [1], that at a critical temperature (T_c_) and pressure (p_c_), two-phase gas–liquid coexistence becomes one supercritical mesophase with both gas and liquid states percolating the phase volume in a colloidal state of coexistence. There is no supercritical “continuity of gaseous and liquid states" as originally hypothesized by Andrews [2]. Moreover, the hypothetical concept of a van der Waals ‘critical volume’ at a singular point [3] is non-existent. The percolation lines delimit the existence of subcritical separate gas and liquid phases, at their different respective critical densities, extending to supercritical temperatures to define the supercritical mesophase.

Here, we continue further analysis following previous JETC (Joint European Thermodynamics Conference) presentations [4,5]. The thermodynamic properties of supercritical isotherms have a natural symmetry between gas and liquid corresponding states along the same isotherm [4]. The thermodynamic state function rigidity, ω = (dp/dρ)_T_, where p is pressure, ρ is density, and T is temperature, is the work required to reversibly increase the density of a fluid. Along any isotherm, rigidity decreases with density for the gas phase and increases with density for a liquid. Thermodynamics, therefore, can define a distinction between gas and liquid that extends to supercritical temperatures. For any one-phase system, rigidity is everywhere positive; in any two-phase region, ω = 0. For temperatures above critical coexistence, the rigidity has a constant value in the mesophase that separates the percolation loci which bound the limits of existence of liquid and gas states in the supercritical region. Every state of gas phase has a corresponding complimentary isothermal state on the liquid phase with the same rigidity.

A revised representation of experimental thermodynamic properties, which have improved in accuracy, to 5 or 6 figures, over the decades, was reported at JETC 2017 [5]. Equations-of-state functional forms with continuity of thermodynamic state functions in all derivatives which accommodate a critical-point singularity are fundamentally inappropriate in the vicinity of the critical temperature (T_c_) and pressure (p_c_) and in the supercritical density mid-range between gas- and liquid-like states. An alternative simpler representation of supercritical isotherms using only known physical constants of the fluids is further prima facie evidence for the absence of a critical singularity on Gibbs density surface at T_c_. It also describes the linear state functions of the supercritical mesophase bounded by 3rd order phase transitions, i.e., discontinuities in the third derivatives of Gibbs energy with respect to p or T [5].

When the gaseous and liquid states are in thermodynamic coexistence at subcritical temperatures, there is a positive surface tension and the first-order phase transition is characterized by a two-phase separation region with a latent heat, a change in molar enthalpy at constant temperature, and a change in molar volume at constant pressure. If the surface tension, and hence also interfacial excess Gibbs energy, were to be zero or negative, first-order coexistence would not be possible, as the two states would spontaneously disperse. The experiments of Maas and his colleagues, over the 8-year period 1932–1940, e.g., [6] and other references therein, show that is precisely what is seen to happen at T_c_. When a coexisting two-phase fluid with a meniscus is heated to a critical temperature, on disappearance of the meniscus at and above T_c_, both states can be seen to coexist without gravitational phase separation.

**Figure 1:**
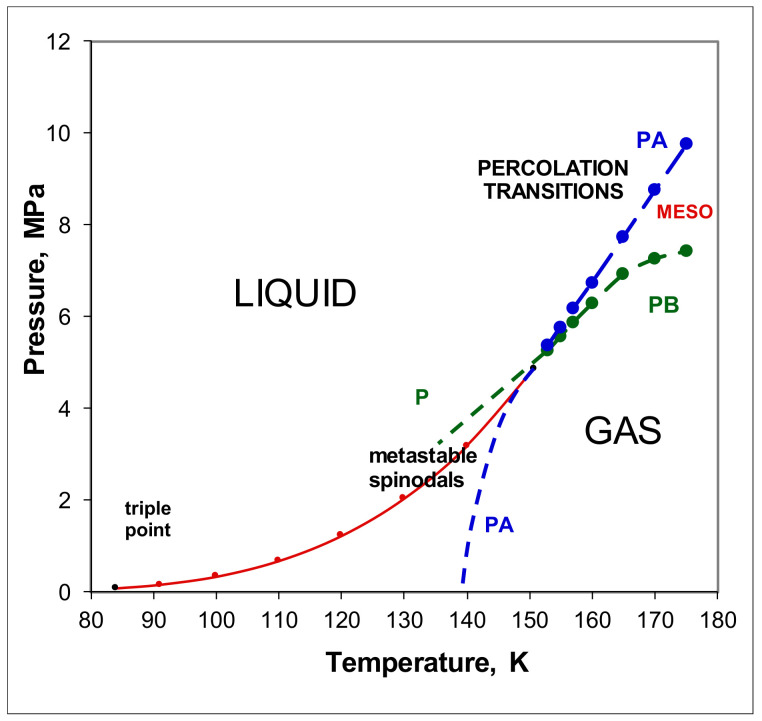
p–T projection of the liquid-gas coexistence line for argon from measurements of Gilgen et al. [6,7]; PA (blue) is the available-volume percolation transition locus and PB (green) is the bonded-cluster percolation transition; the point intersection of PA and PB defines the critical “divide” at T_c_, pc whereupon two phases coexist with different densities; PA and PB continue below T_c_ as metastable spontaneous nucleation loci in the two-phase coexistence region.

A critical point (T_c_,p_c_) is obtained at the intersection of two percolation loci, designated PB and PA, as shown from a reinterpretation of experimental thermodynamic data for liquid argon in Figure 1 [7,8]. One cannot deduce from Figure 1, however, the composition or thermodynamic description of a state point in the mesophase, as there can be no superscrritical lever rule mass balance. Unlike the subcritical two-phase coexistence region whence both gas and liquid coexist at the same T, and p, with only one degree of freedom, the mesophase is a single phase with two states and 2 degrees of freedom (both T and p). Figure 1 shows that along an isotherm, the liquid state limit at PA has a higher pressure than the gas at PB. Likewise, along a supercritical isobar, the limiting gas phase PB has a higher temperature than the liquid state PA. Since, at equilibrium, there can be no gradients in temperature or pressure, there must exist a colloidal state with an interfacial contribution to the stabilization of a two state coexistence. This begs the question “what is the mole fraction composition and thermodynamic description of the coexisting states and the interface between them?”.

**Figure 2:**
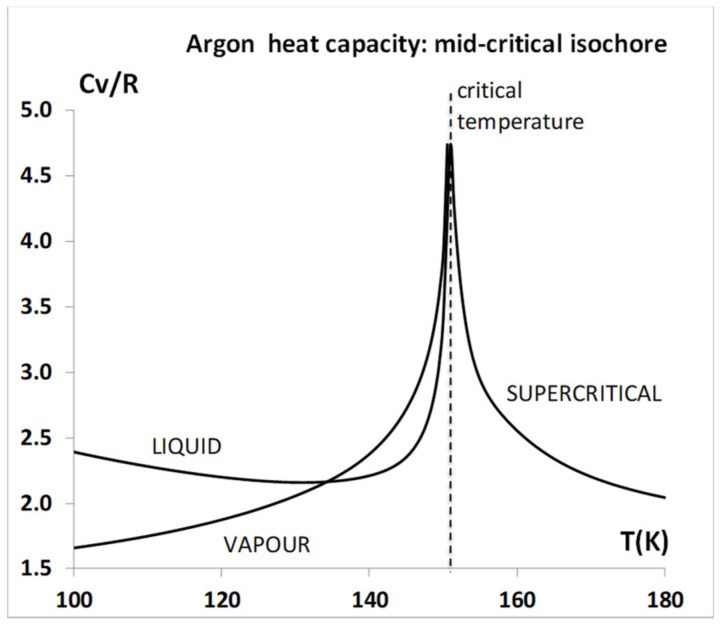
Isochoric heat capacities of argon from NIST (National Institute of Standards and Technology) thermophysical tables [7] along the coexistence lines T < T_c_, and superctitical Cv T > T_c_ (T_c_ – 150.6 K for the mid-critical isochore density = 13.5 mol/L: this is the heat capacity manifestation of hetero-phase fluctuations increasing beginning about 10 K below T_c_.

**Figure 3:**
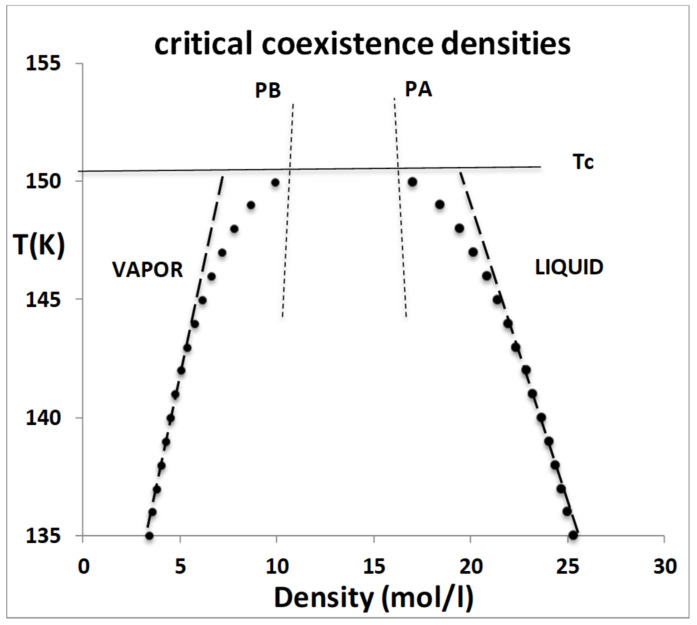
Coexistence curves for near-critical fluid states of argon; the black dots are experimental equilibrium data points from NIST [3] and the dashed lines are the hypothetical homo-phase states defined by a subtraction of hetero-phase fluctuations as described in the text. PB and PA are the percolation transition loci that delineate the existence of equilibrium coexisting gas and liquid phases, respectively.

Accordingly, the motivation for the analysis reported here is to understand the observed experimental thermodynamic state functions at T ≥ T_c_ in terms of the colloidal properties of a mixture of both gaseous and liquid states arising from hetero-phase fluctuations on either side of the percolation transitions that define the mesophase region. Using thermodynamic properties of argon as the exemplary fluid [9], Figure 2 shows that the isochoric heat capacities of the liquid and vapour have normal gas and liquid values (1.5 to 2.5 R) from the triple point temperature (84 K) to around 140 K. Then, at just a few degrees below the critical temperature (Tc = 150.7 K) they begin to increase sharply, peaking at a constant value of around 4.5 R at the critical temperature, and then decreasing again as the fluid is heated into the supercritical region. The isochore density of Figure 2 is in the middle of the two-phase region, as shown in Figure 3 (below).

If the critical point is approached by heating from temperatures below T_c_, i.e., by increasing T at constant volume for the saturated liquid–vapour system, the percolation transitions terminate the coexistence envelope at T_c_, whereupon the surface tension becomes zero. Hetero-phase fluctuations are a well-known extensively studied phenomena [10], and relate to critical properties in other model gas-liquid systems [11]. A colloidal state of the dispersed heterophase in the continuous homophase precedes percolation of both gas and liquid states as they coalesce to mutually permeate the phase volume. In the following sections, we explain the various experimental observations of critical and supercritical fluids by heterophase fluctuations and consider the role of surface tension in this process of stabilisation of the colloidal two-state fluid phases.

## 2. Hetero-Phase Fluctuations

Pre-transition anomalies in thermodynamic properties are well documented and generally explained by heterophase fluctuations, with a long history of experimental investigation and literature discussion [10]. The effect is manifested by a bifurcation of higher-order thermodynamic properties, i.e., higher derivatives of Gibbs energy with respect to temperature or pressure, e.g., heat capacity, and compressibility, respectively, in the vicinity of the transition to the more stable phase. It is based on the idea that the macroscopic transition of a substance from one phase to another begins with an increased presence of small nuclei of the new phase within the host old phase equilibrium state as the transition is approached. These micro-clusters in gas phase, or gaseous-like holes, or ‘bubbles’ in liquid phase, can develop from spontaneous equilibrium density fluctuations which are manifestations of a generalized statistical equilibrium in which they may be seen as dispersed hetero-phase particles, with the host phase considered as quasi-solvent [10].

The isochore showing the anomolous peak in the heat capacity C_v_ in Figure 2 can then be explained by reference to the temperature–density equilibrium coexistence envelope for fluid argon in the vicinity of T_c_, shown in Figure 3. The density corresponding to the isochoric C_v_ values in Figure 2 is 13.5 mol/L, which is directly mid-way between the percolation transitions (PB-gas and PA-liq).

The dashed lines showing a bifurcation of the densities of coexistence of liquid and vapor, which have been added schematically in Figure 3, suggest the formal definition of a concept in the vicinity approaching the percolation loci, a ‘hypothetical homo-phase’. The dashed lines are illustrative of a homo-phase gaseous state with a lower density that the equilibrium state, and a homo-phase liquid with a higher density that the equilibrium liquid, approaching T_c_. These hypothetical non-equilibrium homo-phases can be used to explain what happens at the percolation transitions when T ≥ T_c_.

At T_c_ a two-phase equilibrium fluid with a meniscus, i.e., separating gas from liquid under gravity, is heated beyond T_c_. The gas phase just below T_c_ is a mixture of homo-phase gas at density ρ_h_(gas)_T_ plus homo-phase liquid at density ρ_h_(liquid)_T_, whereas the equilibrium liquid is also a mixture of ρ_h_(liquid)_T_ plus ρ_h_(gas)_T_. This is an identical mixture. Both the equilibrium gas approaching T_c_ and the equilibrium liquid at the same T, are in coexistence at the same T, p, and μ (Gibbs chemical potential). Hence, from the thermodynamic equilibrium identity relating the Gibbs energy difference between alternative states to the equilibrium constant K = exp(−ΔG/RT), we have, where [X] and [1 − X] are the mole fractions of homo-phase liquid and gaseous states, respectively.

ΔG = RT log_e_ ([X]/[1 − X]) (1)

Since there is a symmetry between corresponding gaseous and liquid states on the same isotherm, arising from density fluctuations [4], when both equilibrium states simultaneously percolate the total phase volume at T > T_c_, the equilibrium composition of gas and liquid state densities need not significantly change at T_c_. The meniscus just disappears [6].

### Surface Tension

There must, however, be present a non-zero surface tension stabilizing a nano-scale interface of coexistence of the liquid and gas homo-states, both of which percolate the phase volume in the mesophase. This observation suggests that although the surface tension between equilibrium subcritical coexisting gas and liquid states goes to zero at T_c_, there will remain a positive surface tension at interfaces between the homo-phases in the supercritical mesophase. At present, this hypothetical surface tension may not be thermodynamically definable, nor experimentally accessible, but it stabilizes the colloidal states involved in the percolation process, from equilibrium hetero-phase gas-in-liquid at PA or liquid-in-gas at PB, to the mesophase.

In the subcritical two-phase coexistence region, the surface tension (γ) also characterizes the difference between gas and liquid states in coexistence. It is usually defined as the work required to create an area (A) of plane interface between the two coexisting phases with the same T, p, μ. The excess Gibbs energy of the system due to the presence of any equilibrium surface energy is simply.

G_s_ = A γ (2)

For all subcritical gas–liquid coexisting states in the two-phase region, both G_s_ and γ are positive, whence G_s_ and therefore G_total_ for the whole system is minimized by phase separation. For the equilibrium liquid in coexistence, however, from the triple point to the critical point, the surface tension decreases linearly, with slight non-linearity in the near critical region, as it goes to zero at T_c_. The difference between the rigidities of gaseous and liquid states, notably.

**Figure 4:**
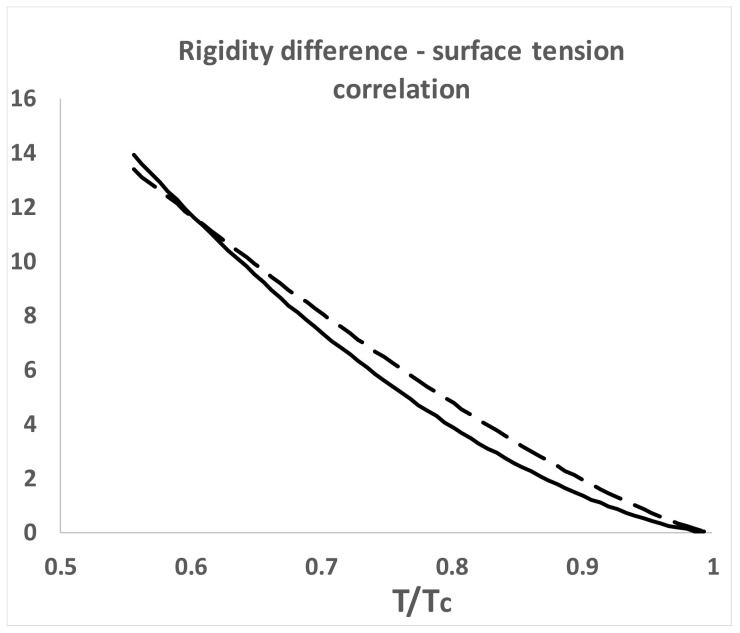
Surface tension (γ) correlation with rigidity difference (Δω) for argon coexisting states from the triple point (84 K) to the critical temperature (T_c_): dashed line is the surface tension in units of MN/m; solid line is the molar rigidity difference between coexisting gas and liquid states in Joules.

Δω = ω (liq) − ω (gas)(3)

correlates with the surface tension (Figure 4). This correlation can be understood: the difference in work required to increase the density of the coexisting states by adding molecules at constant volume is physically equivalent to the work required to create the interface by reducing the density of the liquid and simultaneously increasing the density of the vapor on either side of the Gibbs dividing surface; a line defined within the interface such that the total mass within an arbitrary volume containing the interface remains constant.

Thus, although the surface tension and the rigidity difference correlate and approach zero at T_c_, a surface tension of the coexisting homo-phases, both at T_c_ and in the supercritical range T > T_c_, must exist and remain finite. This suggests the critical state of two coexisting homo-phases extends well into the supercritical region, and probably all the way to the Boyle temperature, as evidenced by the correlation of equation-of-state thermodynamic properties [5].

In the next section, we will re-examine the thermodynamic properties of some supercritical isotherms of argon in the light of this binary-homo-phase co-existence that can describe the thermodynamics of a critical coexistence at T_c_ and in the supercritical region.

## 3. Supercritical Percolation of Hetero-Phase Fluctuations

**Figure 5:**
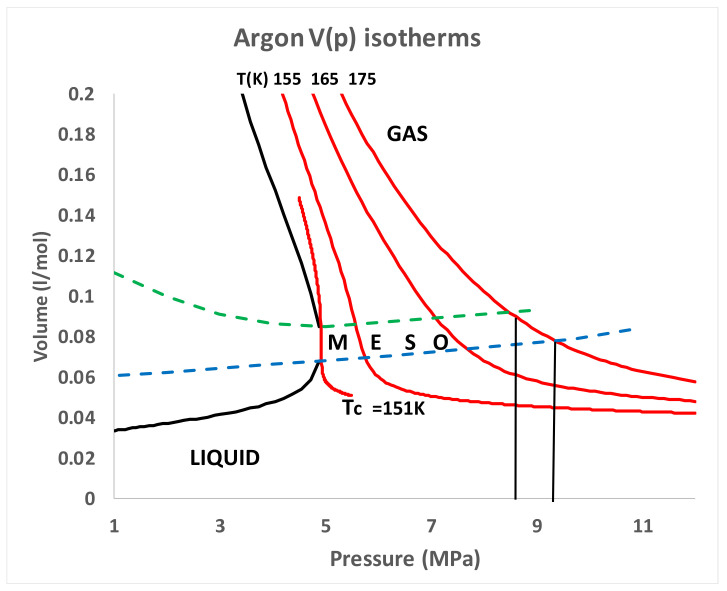
Critical and supercritical V(p) isotherms for argon; the blue and green dashed lines are the percolation loci PA and PB, respectively, obtained as described in reference [4].

Since the original discovery of a critical temperature by Thomas Andrews [2], and subsequent theory and hypothesis of the existence of a “critical volume” by van der Waals [3], there has been a general acceptance of the existence of a singular point. Indeed, since the theoretical physics community declared that there existed a singular point with universal scaling properties in all critical phenomena [12], the hypothesis of van der Waals has been accepted as established as scientific truth, albeit incorrect. This unfortunately has drastically prohibited the scientific experimental and 8theoretical development of the description of gas–liquid thermodynamic equilibria for 50 years. When volume is plotted against pressure (Figure 5) however, one can see that the existence of the supercritical mesophase linear region, and higher-order phase transitions associated with rigidity v. density plots that reveal the essential role of the percolation transitions and the mesophase in the thermodynamic description of gas liquid criticality. There is no van der Waals hypothetical ‘critical volume’ with a scaling singularity on Gibbs ρ(p,T) surface [1,4,5,8,13].

The area between the two vertical lines bounded by the 175 K isotherm in Figure 5 shows the Gibbs energy difference between the gaseous and liquid equilibrium states on either side of the supercritical mesophase. The foremost observation is that it is rather small, less than one-tenth of RT. Thus, the differences in Gibbs energy being so small suggests that within the mesophase, roughly equal mole fractions of liquid and gas states will be present. We will next look at the thermodynamics of a supercritical isotherm of argon, 175 K, to describe the mesophase using the concept of the hypothetical non-equilibrium homo-phase introduced in the previous section.

**Figure 6:**
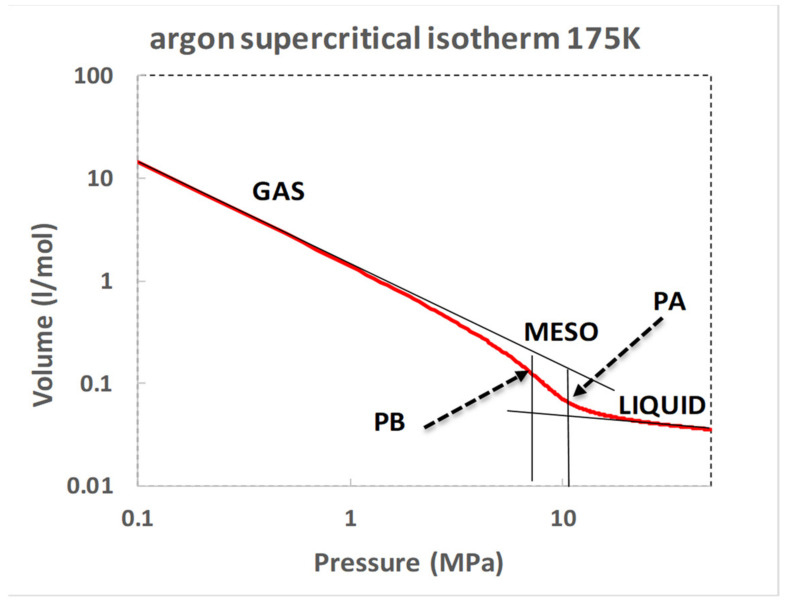
Argon V-p isotherm on log scales showing the regions and effects on the equilibrium molar volumes of hetero-phase fluctuations that precede the percolation transitions PB (gas) and PA (liquid).

Figure 6 shows the V-p argon 175 K isotherm plotted on log scales. The gas isotherm at pressures well below PB and the liquid isotherm at pressures well above PA abbey simple power laws that can be easily parameterised. The regions of hetero-phase fluctuations for both equilibrium gas and liquid sides of the mesophase are identifiable as distinct from the extrapolated hypothetical homo-states. For the 175 K argon isotherm shown, we obtain the results: V_gas_ = 1.387/p^1.025^ and V_liq_ = 0.0822/p^0.215^ where volume V is given in l/mol and the pressure is in MPa.

These very simple but adequate equations-of-state can be used to obtain by extrapolation an approximate estimate of the volumes or densities of the hypothetical homo-phases of gaseous and liquid isotherms in the mesophase.

Assuming the hypothetical non-equilibrium homo-phases can be interpolated into the mesophase regions as a good approximation, we can examine the thermodynamic properties, especially Gibbs energy differences. From the differential equation that combines the first and second laws of thermodynamics.

dG = Vdp − SdT (4)

**Figure 7:**
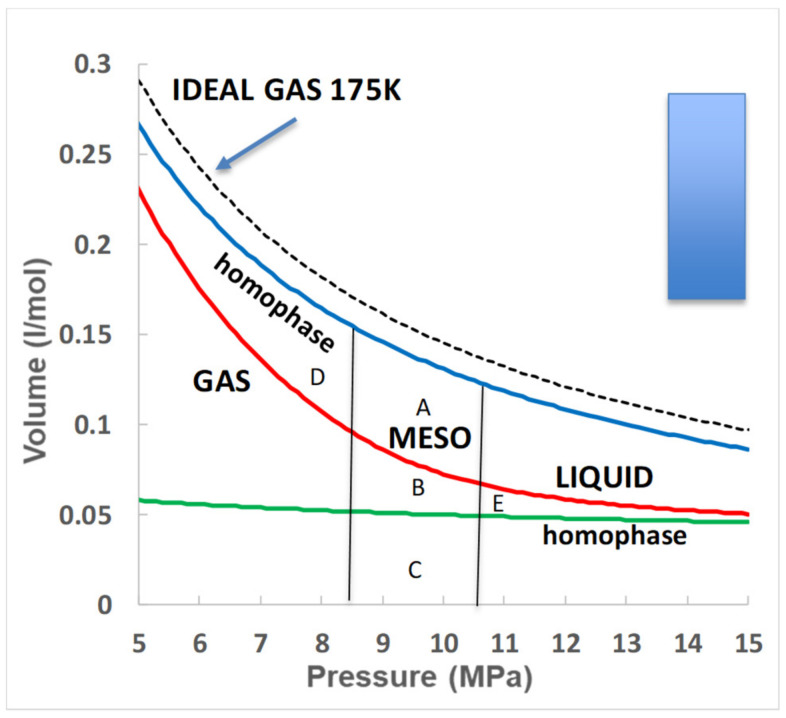
Isotherm data for argon as in Figure 5, but with volume and pressure on linear scales so the area shown ΔG meso corresponds to the difference in Gibbs energy (area B + C) between the gas and liquid states at the respective percolation transition points; the blue and green lines show the hypothetical homo-phase isotherms; the blue area is Gibbs energy G_meso_ 0.0001 MPa·m^3^/mol = 0.06 RT for reference.

Since, along any isotherm dT = 0, we can identify the changes in Gibbs energy, and all other thermodynamic state functions, by simply matching areas in V(p) plots, as illustrated in Figure 7.

The first observation is that the differences and changes in Gibbs energies involved in changing from equilibrium gas to liquid, or homophase gas plus liquid in the mesophase, are all small relative to RT. The blue rectangle of the scale units in Figure 7 is only 0.06 RT. One can immediately see that at the percolation point PA, the difference in Gibbs energy (area D) between the homophase gas and the equilibrium gas is a small fraction of the order of 0.1 RT. We do not have a reversible thermodynamic pathway from the homophase gas to the homophase liquid to obtain a Gibbs energy difference directly, but since these two states coexist in thermodynamic equilibrium, they must have similar chemical potentials, and hence that the Gibbs energy differences between homo-phase gas and liquid in the same single thermodynamic phase is near zero. In fact, if at percolation the chemical potentials are equal, as they must be to coexist at equilibrium, it follows that areas A+ B = B + C, therefore area A = area C, which appears to be approximately true on cursory inspection of Figure 7.

Thus, at the gas bonded-cluster percolation transition PB, we can see that the equilibrium state in the mesophase after percolation is approximately an equimolar mixture of homophase gas and homophase liquid, each with the same chemical potential and hence each phase with the same mole fraction. The gas molar volume is three times the homophase liquid. The same applies to the gas-in-liquid percolation transition PA where the gas homophase at the higher densities is only about three times the homophase liquid. The net result, however, is that Gibbs energy appears to be minimized in the mesophase mixture, notwithstanding a positive interfacial contribution, whence a near equimolar homophase mixture becomes the stable equilibrium thermodynamic state.

## 4. Evidence from Computer Experiments

We have so far only considered experimental evidence for hetero-phase fluctuations in the mesophase colloidal binary-state structure from the thermodynamic properties of argon in Figure 1, Figure 2, Figure 3, Figure 4, Figure 5, Figure 6 and Figure 7. Other liquids, such as the molecular CO_2_ or water, have also been shown to exhibit the characteristic mesophase properties [4,5]. There is a dearth of direct evidence, however, from structural studies such as atomic distribution functions that can be obtained from spectroscopic and scattering experiments. Such compelling evidence, however, has been forthcoming from computer experiments on the critical and supercritical properties of the Lennard-Jones model of simple fluids [13]. We can now re-visit this evidence to essentially corroborate the conclusions regarding a role of hetero-phase fluctuations in preceding the percolation transitions that define the supercritical mesophase.

**Figure 8:**
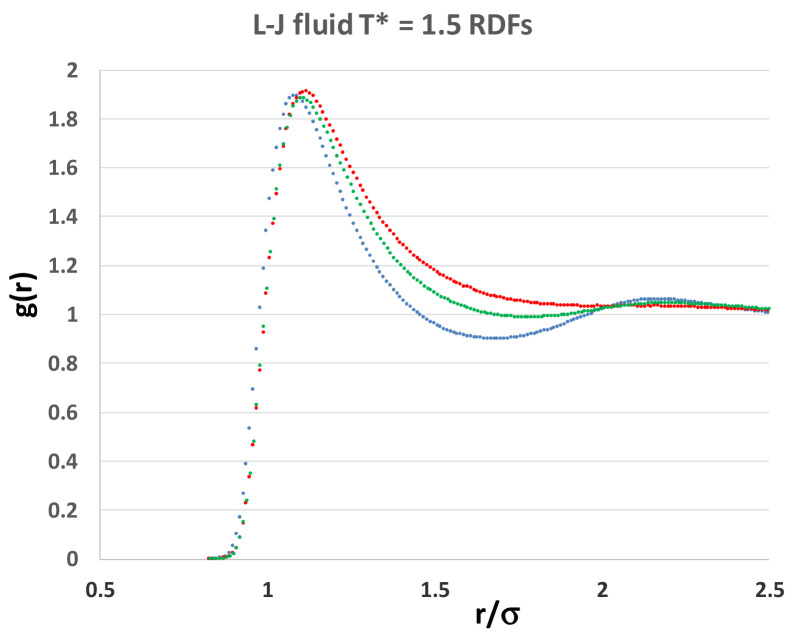
Radial distribution functions of the Lennard-Jones fluid at three densities along the T* = 1.5 supercritical isotherm (T/T_c_ ~ 1.3): the blue line is at the reduced density ρσ^3^ = 0.6 in the quasi-liquid range; the green line is in the mesophase density ρ* = 0.3 and the red line is the gas density ρ* = 0.1. from computations reported in reference [13].

The Lennard Jones fluid was found to have a critical isotherm at kT_c_/ε = 1.3365 ± 0.0005 (where k is Boltzmann’s constant and e is the attractive minimum energy of the L-J pair potential) and several supercritical isotherms were studied in more detail, including the kT/ε = 1.5 isotherm over the whole density range. Highly accurate structural information can be obtained, as illustrated by the radial distribution functions shown in Figure 8. Even though these data points are of very high statistical precision, they do not reveal any information about subtle structural changes that must inevitably accompany the onset of hetero-phase fluctuations, and the third order percolation transitions. We need to examine the normalized pair probabilities (g(r)) at specific distances as a function of density.

**Figure 9:**
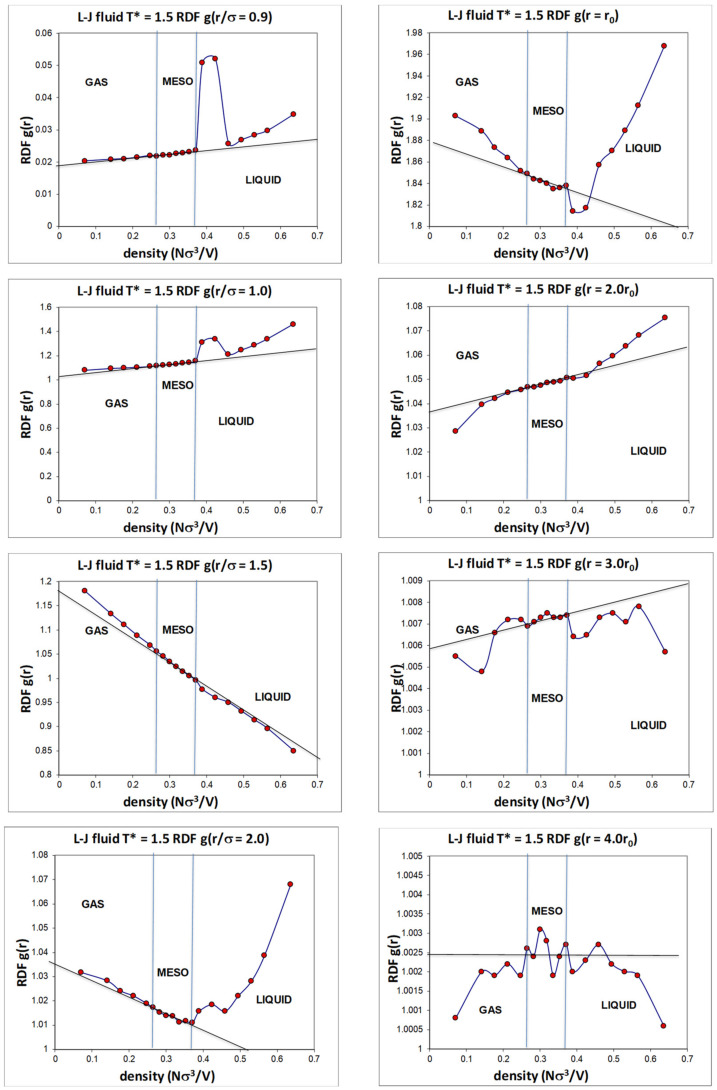
Values of the normalized pair distribution probability function at eight selected intermolecular pair distances ranging from r/σ = 0.9 (**top left**) to 4r_0_ (**bottom right**); σ is the distance of zero pair energy and r_0_ (= 2^1/6^σ) is the distance of zero force; the state parameters along the supercritical isotherm (T* = 1.35); percolation transitions at T_c_* = 1.336 are at reduced densities 0.266 (PB) and 0.376 (PA) [13], as indicated by vertical lines.

If we focus on the density dependence of specific pair distance probabilities, however, we see a different picture. Although we are magnifying miniscule variations in these structural probabilities, the detailed density-dependent structural properties (Figure 9) show unequivocal evidence that there are three distinct state regions, liquid, meso, and gas. More importantly in the present context, we can see the evidence for the hetero-phase fluctuations, which is especially pronounced on the liquid side of the supercritical mesophase at the percolation of available volume (PA), or alternatively of gas-like hetero-phase fluctuations.

An overall analysis of the structural data in Figure 9 shows that in every plot, i.e., at all intermolecular distances from highly repulsive overlap at 0.9σ, to long-range distances of four times the most probable pair distance 4r_o_, there are significant structural differences, as evidenced by the subtle deviations from uniform probability. For distances beyond ~ 4r_o_, when g(r) is very near unity to a fraction of a percent, any differences between the three states become undiscernible within the present statistical uncertainties.

## 5. Conclusions

**Figure 10:**
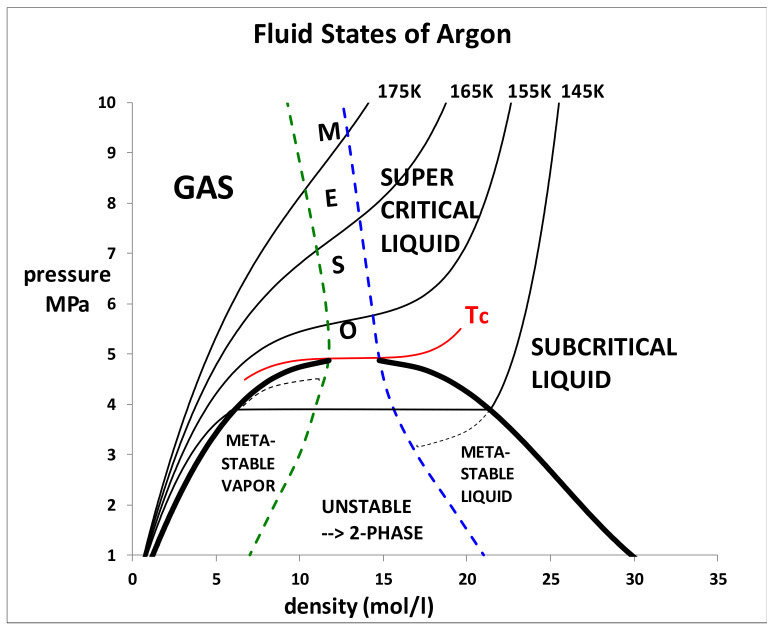
Phenomenological phase diagram of argon showing a subcritical isotherm (145 K) and the percolation loci extending into the two-phase region to define the metastable existence limits of the gaseous and liquid states: the metastable equilibrium branches of the gas and liquid phases terminate at the respective percolation loci (spinodal lines), as shown.

From the foregoing considerations of hetero-phase fluctuations in the role of critical and supercritical condensation of the gas phase, we can conclude that it is both self-consistent, and wholly consistent, with equilibrium and metastable thermodynamic properties of both the supercritical and the subcritical isotherms (Figure 10).

Both gas and liquid states can be extrapolated to some extent into the metastable regions of existence. Figure 10 shows the two-phase coexistence envelope of argon.

The dashed lines showing the metastable branches in Figure 10 support the conclusion that it is the hetero-phase fluctuations of liquid-in gas and gas-in-liquid that percolate to produce spinodal decomposition, which is well-known to be a transient unstable mixture of homo-phase liquid and homo-phase gas with a positive surface tension. Unlike the supercritical “spinodal decomposition” to the mesophase, however, where there is no thermodynamic state with a lower Gibbs energy, the transient mesophase will eventually minimize its Gibbs energy by reducing the interface between the two homo-phases, and returning to the phase-separated gas and liquid with mole fractions determined by the lever rule mass balance, with a meniscus under gravitational force.

Returning to Figure 2, we can now better understand how the increase in C_v_ near to T_c_, which arises from the hetero-phase fluctuations, could be misconstrued as being in support of the misconception that C_v_ may be diverging to infinity at a singularity in accord with universality theory. This misinterpretation evidently gave rise to the original concept of universality as evidenced in the article of Uhlenbeck [12], and also in several other theoretical physics presentations at the same 1965-NBS (now NIST) conference [12]. Further laboratory and computer experimental research are now required to establish to what extent the experimental measurements that appeared to validate the concept of universality gave higher values than the thermodynamic limit, C_v_, perhaps due to a contribution at T_c_ from an onset of a surface tension term on percolation [14,15,16].

It remains a consequence of the second law of thermodynamics, however, i.e., dQ_rev_/T (= entropy) is a state function, and that there can be no divergence of the isochoric heat capacity at any equilibrium state point [17]. One cannot add or remove heat reversibly from a thermodynamic system that can do no external work, without changing T or otherwise violating the second law.
